# ‘Speed advising’ for medical students applying to residency programs: an efficient supplement to traditional advising

**DOI:** 10.3402/meo.v21.31336

**Published:** 2016-04-06

**Authors:** Jillian L. McGrath, Jason J. Bischof, Sarah Greenberger, Daniel J. Bachmann, David P. Way, Diane L. Gorgas, Nicholas E. Kman

**Affiliations:** Department of Emergency Medicine, The Ohio State University College of Medicine, Columbus, OH, USA

**Keywords:** education, undergraduate medical, student advising, education, graduate medical, students, medical

## Abstract

**Background:**

Over time, Residency Match dynamics fluctuate with some specialties experiencing increases in medical student popularity. Academic departments with limited resources must devise methods for coping with increased demand for their specialty. Students perceive traditional programs on Match mechanics as inadequate. Subsequently, faculty are confronted with demands for more personal attention from more students.

**Objectives:**

We developed a strategy for providing specialty-specific residency match advising to large numbers of students.

**Methods:**

The ‘speed-advising’ session (SAS) was developed to address the common questions and concerns that medical students pose during the Match process and to provide advisees with a breadth of faculty perspectives. Two SASs were offered over a 2-week period. After the sessions, students and faculty were surveyed regarding their experience.

**Results:**

Twenty-six students pursued our specialty in the 2015 Match (26 of 234, 11.1%). Twenty-three (89%) participated in the SAS. Seventy-four percent of students (17 of 23) and all faculty completed the post-session survey. Students found the SAS to be informative, helpful and an efficient use of time. Common discussion topics included: *career goals, to which programs and how many to apply*, and *how academic record impacts their likelihood of matching in our specialty*. Students would have preferred more time with each faculty; however, most (77%) conceded that their questions were adequately answered. Faculty-favored speed advising over traditional advising (86%), primarily due to estimated time savings of 7.3 h per faculty member.

**Conclusions:**

In preparing students for the Match, specialty-specific speed advising offers an efficient supplement to traditional advising.

The transition from medical school to residency is a challenging and anxiety-provoking process for medical students. In a very short period of time, students are required to evaluate the strength of their candidacy and select both a specialty that matches their professional interests and a residency program that will most benefit their career. Medical schools provide guidance and support to students throughout medical school (1). However, as students begin to identify their specialty choice, they seek direct guidance and support from physicians in their specialty of interest.

Large-group advising meetings are commonly used to provide general and logistic information to medical students about the National Residency Match Program (NRMP) Match process and how to use the Electronic Residency Application Service (ERAS^®^). These types of informational meetings are most effectively delivered at the third year of medical school when students are beginning the specialty selection process and preparing their applications. Once their choice is made, however, students prefer to meet individually with faculty from their chosen specialty to seek advice and establish relationships.

Fourth-year students feel the need to know more details about the career path they have chosen. They seek advice about the logistics of applying for their specialty of interest. Specifically, they want to know how to: formulate their application, write their personal statement and prepare for interviews (2). Additionally, they seek feedback on their academic record and guidance on which programs they should apply for, whether they should do away-electives, and how many applications they should generate. Furthermore, medical students seek advice from various faculty based on familiarity, educational role or status, and the faculty members own personal background.

Although literature on advising medical students regarding career or specialty selection is prevalent (1, 3) little has been published about career advising for medical students, once their specialty choice has been established (2). Effective post career–identification counseling of students during the Residency Match process requires knowledge of specialty-specific nuances such as: information about the competitiveness of specific training programs of interest, the strength of the student's application, and whether there is a match between the two (4–8). Therefore, medical educators from the specialty of choice play a larger role in helping medical students navigate the application and interview process.

The numbers of students interested in any given specialty fluctuate from year-to-year, and when interest is high, there are increases in demand for faculty time to meet and advise medical students specifically about their specialty. Students will often request meetings with more than one faculty member to compare and contrast the advice they receive. There is also a perception that they need to meet with residency program faculty, particularly if they are interested in matching at their home institution.

Over the past several years, our department has experienced a substantial increase in interest of our specialty and in our residency program (see Fig. 1). This increase has resulted in increased demands for faculty time to meet and advise medical students.

**Fig. 1:**
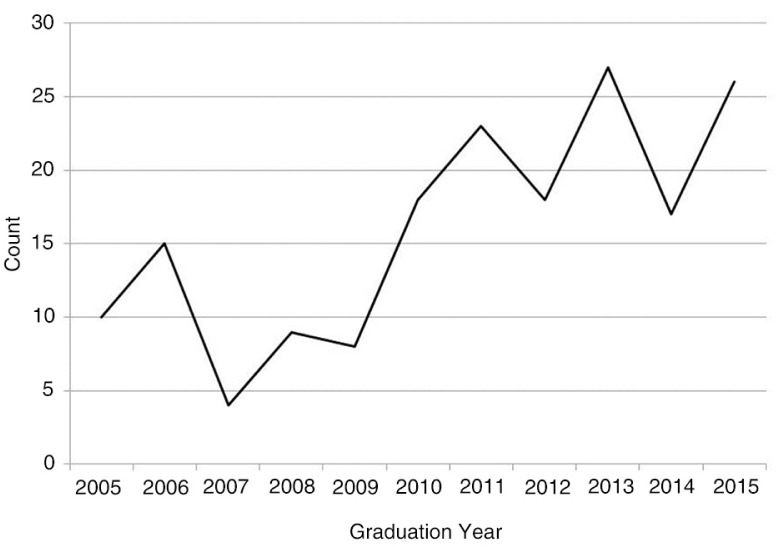
Number of medical school graduates matching in emergency medicine by graduation year at The Ohio State University College of Medicine.

The goal of this project was to devise an effective and efficient alternative for advising medical students who had already selected emergency medicine as their specialty choice. After consideration of the needs of these medical students, we designed and implemented ‘speed-advising’ sessions (SASs) for students pursuing residency in emergency medicine.

## Methods

We recruited all fourth-year medical students who expressed interest in our specialty, emergency medicine to participate in the SAS. We also recruited the emergency medicine faculty whose appointments aligned with administrative roles in medical student or residency programs within the EM department. Faculty were asked to participate on a voluntary basis.

Two 2-h SASs were scheduled and offered 1 week apart during August, 2014. Student advisees were asked to complete a questionnaire in advance of the SAS that detailed their academic record, extracurricular activities, career goals, and residency program preferences. In addition, they were asked to list two or three specific questions that they hoped to have answered during the SAS. Faculty were provided with completed student questionnaires in advance of the SAS.

During the SAS, each student advisee met with up to seven EM education faculty members in succession for8-min intervals. To facilitate quick rotation and minimize time between meetings, we used proximal private offices. At the conclusion of the SASs, students and faculty completed an anonymous survey regarding this advising format.

Both students and faculty were asked whether student's questions were answered, whether there was sufficient time allotted to adequately cover student's questions, and whether they thought the SAS was efficient and informative. Both were also asked about their comfort discussing personal questions during the session, and whether they thought the process was fair and objective. Students were asked whether meeting with multiple faculty members was desirable and faculty were asked whether they prefer this method of advising to traditional methods. Both students and faculty were asked to identify the topics of discussion covered during the SAS. And finally, both students and faculty were asked open-ended questions about what they felt were the strengths and weaknesses of the speed-advising format. Descriptive statistics were tabulated using IBM-SPSS for Windows, Version 22.0 (9). IRB exemption was granted.

## Results

Of 26 students pursuing EM, 23 (89%) participated in the SAS. Of the eight faculty participants, six participated in both sessions, and two participated in one session. The post-session survey was completed by 74% of the student participants (17 of 23) and 100% of the faculty (all eight members). Students met with an average of 6.25 faculty and reported that over half were new to them. Faculty also reported no prior interaction with about 60% of the advisees.

**Fig. 2:**
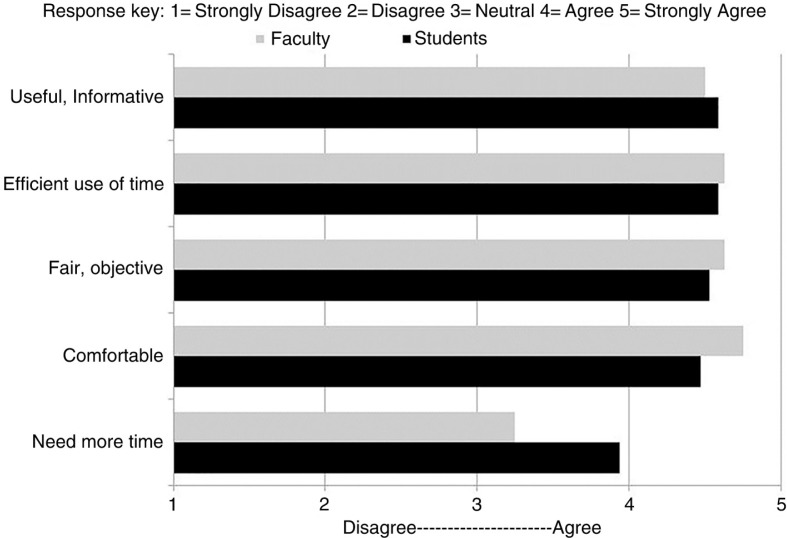
Mean ratings from students and faculty regarding opinions about speed-advising sessions.

All students and faculty found the SAS to be informative and an efficient use of time (see Table 1 and Fig. 2). Almost all faculty and students found the SAS format to be fair and objective (100 and 94%) and were comfortable discussing personal questions (100 and 88%). Students desired longer time intervals with each faculty (71%), but 77% felt their questions were answered adequately. Most faculty members (75%) thought students had all of their questions answered, and few felt the need for longer time intervals with each student (37.5%). Faculty overwhelmingly preferred the speed-advising meeting format to the traditional advising format. Medical students were unanimous in their opinion that meeting multiple faculty in this manner was helpful.

**Table 1:** Post-program survey responses from 8 faculty members and 17 medical student advisees regarding speed-advising sessions

		Faculty			Students		
		
Item		Mn	Std Dev	Pct Agreement*	Mn	Std Dev	Pct Agreement*
1.	I found the speed-advising session to be informative	4.50	0.54	100	4.59	0.51	100
2.	I found the speed-advising session to be an efficient use of my time	4.63	0.52	100	4.59	0.51	100
3.	I found the speed-advising session to be fair and objective	4.63	0.52	100	4.53	0.62	94.1
4.	F: I was comfortable answering student's personal questions/S: I was comfortable asking personal questions	4.75	0.46	100	4.47	0.87	88.2
5.	F: Longer interactions are needed/S: I still need more time with individual faculty	3.25	1.28	37.5	3.94	1.03	70.6
6.	F: Overall, I think the students questions were answered/S: My questions were adequately answered	3.75	1.17	75.0	4.23	0.83	77.0
7.	F: I prefer this method of advising to individually scheduled meetings (20–30 min per student)	4.63	0.74	87.5			
8.	S: I found it helpful to meet with multiple faculty members to discuss matching in EM				4.59	0.51	100

Likert response options were used and coded: 1=Strongly Disagree, 2=Disagree, 3=Neutral, 4=Agree, 5=Strongly Agree.

* Pct Agreement is the percentage who replied with Strongly Agree or Agree.

F=faculty members, S= students, Mn= mean, Std Dev=standard deviation.

The most common discussion topics identified by both faculty and students included: to which programs and how many to apply. More than half the faculty said that application blemishes or problems were a common topic, while only one student agreed. Students also reported discussing: likelihood of matching in EM, standardized letters of evaluation, grades, United States Medical Licensing Examination (USMLE) scores, and career goals less frequently than did faculty (Table 2). The least common discussion topic identified by both faculty and students was non-clinical or extracurricular activities.

**Table 2:** Number (and percentage) of question topics that arose during speed-advising sessions as reported by 8 faculty members and 15 medical students

Topics or questions		Faculty (*n*=8)	Student (*n*=15)
1.	USMLE scores	4 (50.0)	5 (33.3)
2.	Grades	4 (50.0)	5 (33.3)
3.	To which programs should I apply?	8 (100)	13 (86.7)
4.	To how many programs should I apply?	7 (87.5)	12 (80.0)
5.	Likelihood of matching in EM?	3 (37.5)	6 (40.0)
6.	Non-clinical activities?	2 (25.0)	1 (6.7)
7.	Future career goals?	3 (37.5)	5 (33.3)
8.	Do you see any problems or blemishes on my application?	5 (62.5)	1 (6.7)
9.	What about my standardized letters of evaluation?	4 (50.0)	6 (40.0)
10.	Other question topics	0 (0)	1 (6.7)

Faculty comments centered around ‘time’ as the best feature of the SAS format. They liked the efficiency and the opportunity to meet more students. Student comments centered around the value of meeting with multiple faculty as the best feature of the SAS format because they valued obtaining a wider range of opinions.

The faculty suggested that to improve the session, each faculty should be assigned specific topics to discuss with every student they met so that redundancy was eliminated. Students suggested that they needed more time with each faculty member. Some felt that meeting with seven faculty members was too many and that more time with fewer faculty would be preferred.

## Discussion

### Student experience

Students found the SAS to be informative and efficient. They valued advice from multiple faculty and felt their questions were adequately answered, despite a desire for more time with each faculty. Salkowitz maintains that there is both analytic and anecdotal evidence showing the value in trying to explain the attitudes of individuals and groups according to their generational outlook (10). Millennial learners are generally defined as students born from the early 1980s to the early 2000s. This represents the majority of current senior medical students seeking advice about residency training programs. Some characteristics of this generation are the need for speed and innovation and an expectation of others’ investment in their careers (11). The SAS addresses both of these needs for this generation of learners. Further studies may identify what students would consider an optimal time interval with each faculty member (i.e., a few more minutes vs. longer meetings approaching the traditional advising format).

Most strikingly, SAS broadened the students’ advising network by interacting with many new faculty members (students met with an average of 3.7 unfamiliar faculty). Many institutions use single advisor systems, employing either the program director or the clerkship director in this role (6). This can, however, create an inherent internal conflict of the program director in the process of advising. A study by Miller in 2004 demonstrated less confidence in the student when the advisor sat on the residency selection committee (7). If a dual advisor system is instead employed (program director and medical student advisor), studies have shown low levels of congruity of opinions between these two groups (8). Broadening and diversifying the pool of advisors might help to address both of these issues.

### Faculty experience

Faculty advisors overwhelmingly preferred SAS to traditional advisor meetings. Programs have become larger over the past decades, placing more responsibility of managing higher volumes of students on program directors. This is compounded by students seeking the opinions of multiple faculty, including assistant or associate program directors, medical student or clerkship directors, core education faculty, advisors, etc. Faculty identified significant time savings that are multiplied depending on the number of students requesting individual advising meetings. By our estimates the SAS required 253 min (11 min per student including 3-min pre-session form review and the 8-min meeting) vs. traditional advising which would have required 690 min (30 min per student). Several faculty suggested that each faculty advisor should be assigned certain topics from those delineated in Table 1 in order to ensure comprehensive coverage and avoidance of redundancy.

### Limitations

This study took place in a single department in a single academic center with limited sample size. Furthermore, the effect of SAS on long-term outcomes over time has yet to be evaluated. All students who participated in the SAS during this match cycle, however, successfully matched to a residency position in our specialty, emergency medicine or emergency medicine–internal medicine program.

## Conclusions

Although optimal structure and time allotment should continue to be explored, speed-advising sessions allow students to have efficient interaction with multiple advisors while addressing individual concerns about matching in residency. This is a novel advising approach that we believe should be generalizable to other medical specialties and institutions.
